# Different Expression Levels of DLK2 Inhibit NOTCH Signaling and Inversely Modulate MDA-MB-231 Breast Cancer Tumor Growth In Vivo

**DOI:** 10.3390/ijms23031554

**Published:** 2022-01-29

**Authors:** Ana-Isabel Naranjo, María-Julia González-Gómez, Victoriano Baladrón, Jorge Laborda, María-Luisa Nueda

**Affiliations:** 1 Biochemistry and Molecular Biology Branch, Medical School/CRIB/Biomedicine Unit, Department of Inorganic and Organic Chemistry and Biochemistry, University of Castilla-La Mancha (UCLM)/CSIC, 02008 Albacete, Spain; Anaisabel.naranjo@hotmail.com (A.-I.N.); Victoriano.Baladron@uclm.es (V.B.); 2 Biochemistry and Molecular Biology Branch, Higher Technical School of Agricultural and Forestry Engineering/CRIB/Biomedicine Unit, Department of Inorganic and Organic Chemistry and Biochemistry, University of Castilla-La Mancha (UCLM)/CSIC, 02008 Albacete, Spain; MariaJulia.Gonzalez@uclm.es; 3 Biochemistry and Molecular Biology Branch, School of Pharmacy/CRIB/Biomedicine Unit, Department of Inorganic and Organic Chemistry and Biochemistry, University of Castilla-La Mancha (UCLM)/CSIC, 02008 Albacete, Spain

**Keywords:** DLK2, NOTCH signaling, MDA-MB-231 cells, kinase, tumor growth in vivo

## Abstract

NOTCH signaling is implicated in the development of breast cancer tumors. DLK2, a non-canonical inhibitor of NOTCH signaling, was previously shown to be involved in skin and breast cancer. In this work, we studied whether different levels of DLK2 expression influenced the breast cancer characteristics of MDA-MB-231 cells. We found that DLK2 overexpression inhibited NOTCH activation in a dose-dependent manner. Moreover, depending on the level of inhibition of NOTCH1 activation generated by different levels of DLK2 expression, cell proliferation, cell cycle dynamics, cell apoptosis, cell migration, and tumor growth in vivo were affected in opposite directions. Low levels of DLK2 expression produced a slight inhibition of NOTCH1 activation, and enhanced MDA-MB-231 cell invasion in vitro and cell proliferation both in vitro and in vivo. In contrast, MDA-MB-231 cells expressing elevated levels of DLK2 showed a strong inhibition of NOTCH1 activation, decreased cell proliferation, increased cell apoptosis, and were unable to generate tumors in vivo. In addition, DLK2 expression levels also affected some members of other cell signaling pathways implicated in cancer, such as ERK1/2 MAPK, AKT, and rpS6 kinases. Our data support an important role of DLK2 as a protein that can finely regulate NOTCH signaling and affect the tumor properties and growth dynamics of MDA-MB-231 breast cancer cells.

## 1. Introduction

In addition to its pivotal role during embryonic development and tissue homeostasis in adult life, NOTCH signaling is implicated in the development of numerous types of tumors. It is now accepted that NOTCH receptors may act both as oncogenes and as tumor suppressor proteins, depending on the cellular context [1,2,3]. The important role of NOTCH signaling in breast cancer development was established more than two decades ago with the discovery that the mouse *Int3* oncogene, a truncated NOTCH4 protein corresponding to its active intracellular region (NICD4), was implicated in mammary tumors [4]. It is known that NOTCH signaling is aberrantly activated in breast cancer cell lines and primary cell samples. The overexpression of NOTCH receptors and ligands was reported in breast tumors, correlating with poorer patient prognosis. The inhibition of NOTCH signaling was shown to consistently suppress cell proliferation, invasiveness and migration, as well as the development and progression of breast tumors, which is of great importance for therapeutic targeting considerations [5].

Numerous studies established DLK1 (Delta-like homolog 1), a non-canonical ligand of NOTCH receptors, as an inhibitor of NOTCH signaling, including various works published by our group [6,7,8,9,10,11,12,13]. In a previous work, we studied the role of DLK1 in the tumor properties of MDA-MB-231 breast cancer cells [10]. We confirmed that human DLK1 inhibited NOTCH1 activation in these cells, and we revealed that their proliferation rate and invasion capabilities depended upon the level of NOTCH1 activation as negatively regulated by the level of DLK1 expression. A significant decrease in MDA-MB-231 cell proliferation and invasion was associated with high levels of DLK1 expression, which produced a significant inhibition of NOTCH1 activation. However, low levels of DLK1 expression produced low levels of inhibition of NOTCH1 activation, which unexpectedly caused an enhanced in vitro MDA-MB-231 cell invasion and increased cell proliferation both in vitro and in vivo. Therefore, NOTCH1 activation levels, as negatively regulated by DLK1, appeared critical for the function of this receptor as an oncogenic or as a tumor suppressor protein in MDA-MB-231 cells.

DLK2 (Delta-like homolog 2) is a protein highly homologous to DLK1 [14]. As with DLK1, DLK2 was shown to interact with NOTCH receptors and function as another non-canonical inhibitor of NOTCH signaling [8,11,13]. It was reported that, similarly to DLK1, DLK2 affects the growth of SK-MEL-2 melanoma cells, and the nature of its effects depends upon DLK2 expression levels and the degree of inhibition of NOTCH1 activation [9]. Recently, DLK2 was described as overexpressed in lethal prostate cancers, uveal melanoma, breast cancer cell lines and in patient tissues spanning three breast cancer subtypes (Luminal A, Luminal B, and Triple Negative) [15,16,17,18].

In this work, we explored the effects of different DLK2 expression levels on the characteristics of MDA-MB-231 breast cancer cells. We found that increased DLK2 expression levels led to the increased inhibition of the NOTCH1 receptor. However, only elevated levels of the inhibition of NOTCH1 activation, achieved by elevated levels of DLK2 expression, led to decreased cell growth. Cell cycle dynamics and apoptosis were also affected by DLK2 expression levels in opposite directions. The invasive properties of these cells were also affected. Thus, low levels of DLK2 expression led to the increased invasive properties of MDA-MB-231 cells, whereas high levels of DLK2 expression did not significantly modify them. These invasive alterations were associated with changes in the expression levels of cell adhesion proteins, such as E-cadherin and N-cadherin, involved in epidermal-to-mesenchymal transition (EMT) and carcinogenesis [19]. In addition, DLK2 expression levels also affected members of other cell signaling pathways, including ERK1/2 MAPK (mitogen-activated protein kinase/extracellular signal-regulated kinase), AKT (protein kinase B), and rpS6 (ribosomal protein s6) kinases. Finally, MDA-MB-231 cells expressing high levels of DLK2 were unable to generate tumors in vivo in a nude mouse model, an effect that was not observed in control cells or cells expressing low levels of DLK2. Overall, these data support an important and complex role of DLK2 in the control of NOTCH signaling and in the tumorigenic properties and growth dynamics of MDA-MB-231 cells.

## 2. Results

### 2.1. Generation of Transfected MDA-MB-231 Cells Stably Expressing Different Levels of Human DLK2 Protein

MDA-MB-231 cells, a triple-negative breast cancer cell line, display representative epithelial-to-mesenchymal transition (EMT) associated with breast cancer metastasis [20]. We selected this cell line for our studies because it represents an aggressive and fast-growing type of breast cancer, capable of generating metastasis in vivo [21]. In addition, our previous analyses confirmed that MDA-MB-231 cells do not express DLK1 [10], although they do express DLK2. We estimate that this property is suitable for studying the potential effects of the forced overexpression of DLK2 on triple-negative breast cancer cell growth without interfering with the expression of its homolog, DLK1.

**Figure 1:**
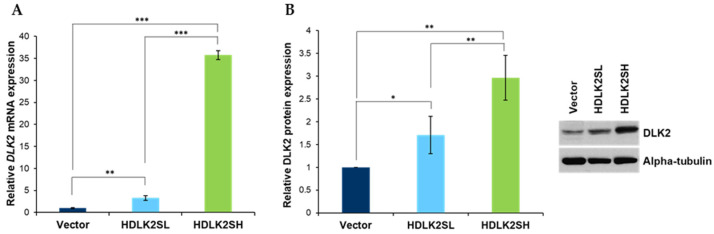
**Generation of MDA-MB-231 cells with different levels of DLK2 expression**. Cells were transfected with the DLK2 expression plasmid (HDLK2S) or the empty vector. Different transfected pools were obtained, and the expression levels of mRNA and protein were analyzed. (**A**) Analysis of *DLK2* mRNA expression levels by RT-qPCR. (**B**) Analysis of DLK2 expression by Western blot and densitometric analysis. Two pools showing significantly different *DLK2* mRNA and DLK2 protein expression levels, termed HDLK2SL (low expression level) and HDLK2SH (high expression level), were selected and used in subsequent assays. * *p* ≤ 0.05, ** *p* ≤ 0.01, *** *p* ≤ 0.001 (Student’s *t*-test results relative to empty vector control cells).

MDA-MB-231 cells with different levels of DLK2 expression were obtained as described in Materials and Methods by transfecting several plates of cells with the HDLK2S plasmid or the empty vector. After each transfection, cells able to grow in a selective medium were pooled, and their *DLK2* mRNA and DLK2 protein expression levels were analyzed. By these procedures, we ended up selecting two different pools of MDA-MB-231 cells that expressed low or high levels of *DLK2* mRNA, compared with empty-vector cells, used as a control, as determined by RT-qPCR. These pools of cells were named HDLK2SL and HDLK2SH, respectively (Figure 1A).

We also performed Western blot assays to determine DLK2 protein expression levels in the HDLK2SL and HDLK2SH cell pools compared with empty-vector cells. Images were quantified by densitometry. As expected, DLK2 levels were lower in HDLK2SL cells than in HDLK2SH cells. The differences between both pools, as well as between each pool and the pool of cells transfected with the empty vector, were statistically significant (Figure 1B).

### 2.2. Different Levels of DLK2 Expression Inversely Modulate the Growth of MDA-MB-231 Cells

**Figure 2:**
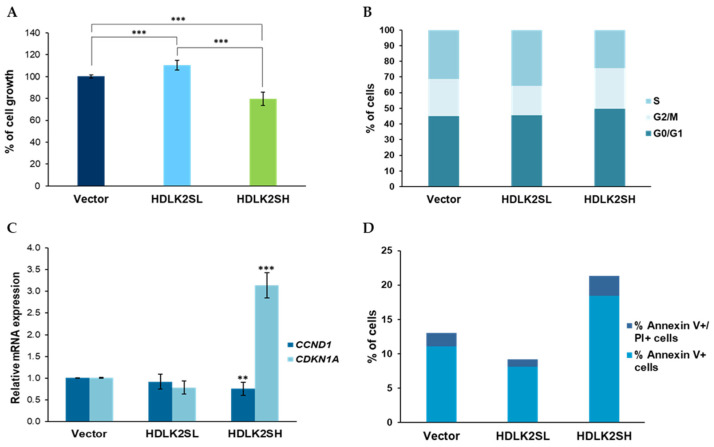
**Overexpression of human DLK2 modulates cell proliferation and apoptosis.** (**A**) The growth rates of HDLK2SL and HDLK2SH cell pools were analyzed by MTT assays. The graph shows the percentage of cell growth (mean ± SD) of the indicated transfectants compared to that of empty-vector cells (adjusted to 100%) after 8 days of culture. (**B**) Cell cycle distribution analysis of HDLK2SL and HDLK2H cells by flow cytometry. (**C**) Relative mRNA levels of *CCND1* and *CDKN1A*, determined by RT-qPCR. (**D**) Percentage of each transfected cell line stained with annexin V and propidium iodide (PI). Details on how the different assays were performed are described in Material and Methods section. ** *p* ≤ 0.01, *** *p* ≤ 0.001 (Student’s *t*-test results relative to empty-vector cells).

To analyze how different DLK2 expression levels affected the growth kinetics of MDA-MB-231 cells in vitro, the two pools of DLK2-expressing cells were grown in a culture medium for several days and their cell numbers were estimated at various time points by an MTT assay (see Materials and Methods). The results showed that cell growth was strongly dependent upon DLK2 expression levels. HDLK2SL cells showed a higher proliferation rate compared with that of cells transfected with an empty vector. However, HDLK2SH cells grew at a lower rate compared to cells transfected with an empty vector (Figure 2A). Overall, after eight days in culture, HDLK2SL and HDLK2H cells had grown at a very significantly different rate, the cells with higher levels of DLK2 expression showing a slower growth rate.

To try to uncover the reasons of these remarkable differences between the two cell pools, we first studied the cell distribution in the different phases of the cell cycle by flow cytometry. We observed that a higher percentage of HDLK2SH cells were in the G0 phase of the cell cycle, compared to HDLK2SL or empty-vector cells (Figure 2B), suggesting that a decreased cell cycle rate was associated with higher levels of DLK2 expression. Consistent with this, a lower percentage of HDLK2SH cells was detected in the S phase, with a concomitant increase in the percentage of cells in the G2/M phase [22].

Using RT-qPCR, we also determined the relative mRNA levels of *CCND1* (cyclinD1) and *CDKN1A* (p21, Cip1), two proteins involved in cell cycle progression [23]. No significant differences were observed between HDLK2SL cells and empty-vector cells in the relative expression levels of *CCND1* or *CDKN1A* mRNAs. However, HDLK2SH cells showed significantly higher levels of *CDKN1A* and lower levels of *CCND1* (Figure 2C), which may help to explain the slow cell growth rate of these cells compared to that of HDLK2SL or empty-vector cells.

Increased apoptosis could also be a factor affecting the different overall cell growth rates observed in cells with high and low DLK2 protein levels. To study this aspect, we determined annexin V/ propidium iodide (PI) double staining by flow cytometry. Annexin V and propidium iodide staining was more intense in HDLK2SH cells and less intense in HDLK2SL cells compared to empty-vector cells (Figure 2D). Thus, apoptosis also appeared to be affected by DLK2 expression levels consistent with the observed effects on cell growth.

### 2.3. Invasion Features of DLK2-Overexpressing MDA-MB-231 Cells

**Figure 3:**
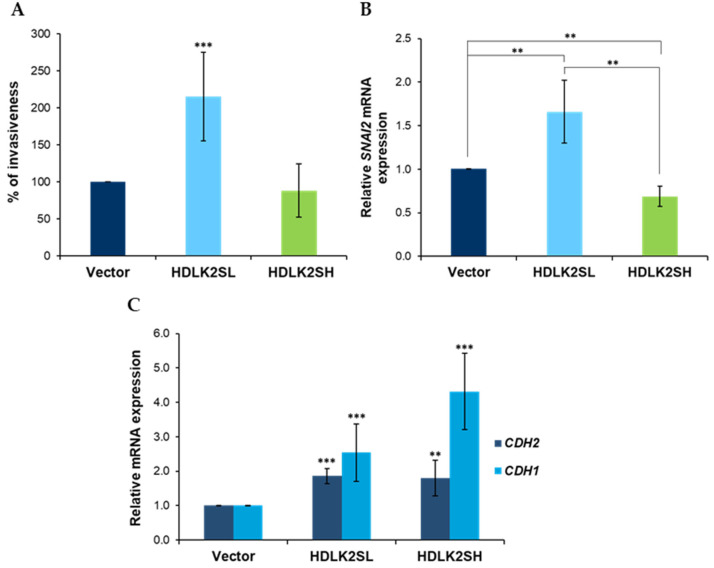
**Invasion features of MDA-MB-231 cells overexpressing DLK2.** (**A**) HDLK2SL cells, expressing low levels of DLK2 showed a significantly increased invasion capacity compared with empty vector or HDLK2SH cells. (**B**) Expression levels of the transcription factor *SNAI2*, involved in the epithelial-to-mesenchymal transition, as determined by RT-qPCR. (**C**) Relative expression levels of *CDH1* and *CDH2*, as determined by RT-qPCR. ** *p* ≤ 0.01, *** *p* ≤ 0.001 (Student’s *t*-test results relative to empty-vector cells).

The increased malignancy of tumor cells is related to their ability to establish metastasis to distant organs or tissues. To achieve this, the cells must leave the original tumor and migrate. To determine whether DLK2 overexpression could affect the migration ability of MDA-MB-231 cells, in vitro invasion assays were performed as described in Materials and Methods section. Figure 3A shows that DLK2 overexpression levels altered the invasion potential of these cells. HDLK2SL cells showed a significant increase in invasiveness, whereas HDLK2SH cells showed no significant changes in this property compared to empty-vector cells.

The invasiveness of breast cancer cells depends on the activity of transcription factors that modulate the expression of cell adhesion proteins, such as E-cadherin, encoded by the *CDH1* gene and N-cadherin, encoded by the *CDH2* gene, and the epithelial-to-mesenchymal transition. The zinc finger protein Slug, encoded by the *SNAI2* gene [24,25], is one of these transcription factors. This gene is upregulated by NOTCH activation in colorectal cancer [26] and in MDA-MB-231 cells [27]. Our transfected cells showed that *SNAI2* expression levels were affected by DLK2 expression levels. HDLK2SL cells showed a significant increase, whereas HDLK2SH cells showed a significant decrease, in *SNAI2* expression levels (Figure 3B).

As expected, the expression levels of *CDH1* and *CDH2* were affected by the changes in *SNAI2* expression levels, which are caused by the different levels of DLK2 expression. Both HDLK2SL and HDLK2SH cells showed an increase in *CDH1* and *CDH2* mRNA expression levels. However, *CDH1* expression levels were higher for HDLK2SH cells (Figure 3C).

### 2.4. DLK2 Inhibited NOTCH Activation in MDA-MB-231 Cells in a Dose-Dependent Manner

**Figure 4:**
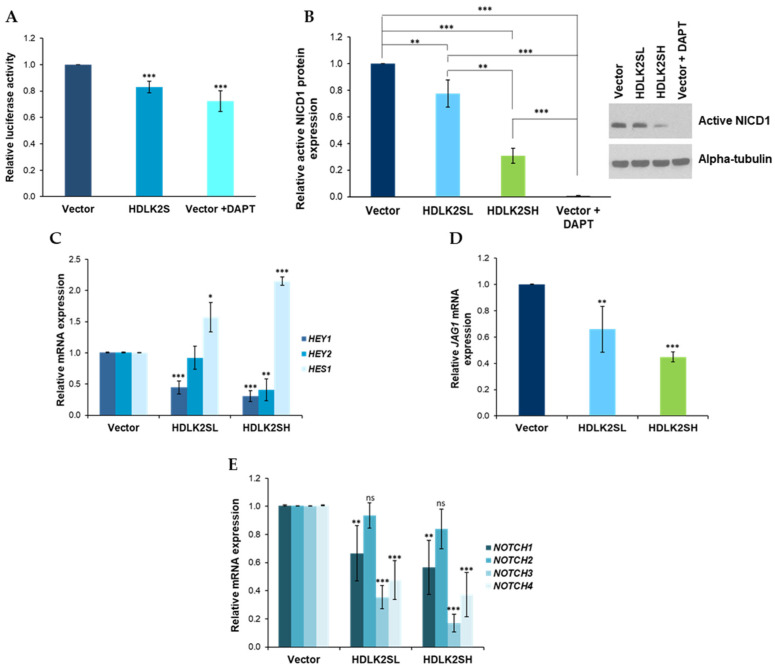
**DLK2 inhibits NOTCH activation and signaling in MDA-MB-231 cells in a dose-dependent manner.** (**A**) Analysis of the overall NOTCH signaling status in MDA-MB-231 cells transiently transfected with the empty vector or the HDLK2S expression plasmid as determined by luciferase assays. Treatment of empty-vector cells with the NOTCH inhibitor DAPT was used as a positive control. (**B**) Western blot and densitometric analysis of active NICD1 expression in HDLK2SL- and HDL2SH-stable transfectant cells. As before, treatment of empty-vector cells with the NOTCH inhibitor DAPT was used as a positive control. (**C**) Expression analysis of NOTCH target genes *HES1*, *HEY1* and *HEY2* in HDLK2SL- and HDLK2SH-stable transfectant cells by RT-qPCR. (**D**) RT-qPCR analysis of *JAG1* expression level in HDLK2SL- and HDLK2SH-stable transfectant cells. (**E**) RT-qPCR analysis of *NOTCH1* to *NOTCH4* mRNA expression levels in HDLK2SL- and HDLK2SH-stable transfectant cells. Details of the procedure are described in Materials and Methods section. * *p* ≤ 0.05, ** *p* ≤ 0.01, *** *p* ≤ 0.001 (Student’s *t*-test results relative to empty-vector cells).

In previous works, we showed that DLK2 functions as an inhibitor of NOTCH signaling in mouse preadipose cells [8,11,13] and in human melanoma cells [9]. The level of NOTCH1 signaling greatly affected the proliferation rate of melanoma cells in a non-linear fashion. For these reasons, we hypothesized that DLK2 might be acting by a similar mechanism on MDA-MB-231 cells. The overall state of NOTCH signaling depends not only on NOTCH1 but also on the activation of the other three NOTCH receptors (NOTCH 2-4) expressed by MDA-MB-231 cells [10]. To assess this, we first estimated the global levels of NOTCH signaling in MDA-MB-231 cells transiently transfected with the HDLK2S plasmid by determining the activity of a luciferase reporter gene under the control of a NOTCH-dependent promoter that possesses binding sites for the CSL/RBP-Jk factor. We observed a significant decrease in luciferase reporter gene activity in MDA-MB-231 cells transiently transfected with the HDLK2S plasmid compared with cells transiently transfected with the empty vector (Figure 4A). The degree of inhibition was comparable to that obtained when cells transiently transfected with an empty vector were treated with the NOTCH inhibitor DAPT at a concentration of 10 μM for 24 h. Thus, this result confirmed a global NOTCH inhibitory role of DLK2 on NOTCH activation and signaling.

Next, we analyzed NOTCH activation levels in our two pools of DLK2-transfected cells. First, NOTCH1 activation levels were determined by Western blot by using an antibody that specifically detects the active intracellular domain of NOTCH1 (NICD1). Both HDLK2SL and HDLK2SH cell pools showed a significant decrease in NICD1 levels, indicating lower levels of NOTCH1 activation. The degree of inhibition was proportional to the level of DLK2 expression, and HDLK2SH cells showed a significant and stronger decrease in NOTCH1 activation levels than HDLK2SL cells, which also showed a significant inhibition of NOTCH1 activation compared to empty-vector cells (Figure 4B). As a control, we treated empty-vector cells with the gamma-secretase inhibitor DAPT (10 μM) for 24 h, a treatment known to suppress NOTCH receptors activation (Figure 4B).

We also studied the expression levels of *HEY1*, *HEY2* and *HES1*, three NOTCH receptors target genes (Figure 4C) [28]. As expected, the expression levels of *HEY1* and *HEY2* correlated with the levels of NOTCH1 activation shown by cells with different levels of DLK2 expression. Thus, both HDLK2SL and HDLK2SH cells showed lower *HEY1* and *HEY2* expression levels than empty-vector cells, although this decrease was not significant for *HEY2* in the case of HDLK2SL cells. However, *HES1* expression levels showed an increase in both pools of transfected cells, rather than the expected decrease, and the increase was greater in HDLK2SH cells. We do not have an explanation for these data; perhaps it is possible that changes in the stoichiometry of NOTCH 1-4 receptor activation and canonical ligand expression caused by DLK2 expression levels may lead to increased expression of some NOTCH target genes but to the inhibition of others.

To further study this possibility, we decided to analyze the expression levels of the *JAG1*, which encodes for Jagged1, one of the most important canonical NOTCH receptor ligands in breast cancer [27], as well as the expression levels of the four NOTCH receptor genes in our DLK2-overexpressing cells. We also analyzed the expression of the *JAG2* gene, but it was not expressed by these cells. We hypothesized that if DLK2 preferentially inhibited one or a few, but not all, NOTCH receptors, this inhibition might somehow be compensated by the increased expression levels of the Jagged1 ligand, or by the increased expression levels of at least one of the *NOTCH* receptors, which could lead to the observed increase in *HES1* expression, despite lower overall levels of NOTCH activation. However, this was not what we observed. The expression level of *JAG1* decreased as DLK2 expression levels increased (Figure 4D), indicating that the direct inhibition of NOTCH activation caused by DLK2 could also be associated with an indirect inhibition caused by decreased expression levels of *JAG1* ligand. Moreover, all *NOTCH* receptor genes, except *NOTCH2*, showed a significant decrease in their expression levels in both HDLK2SL and HDLK2SH cells (Figure 4E). The most significant reduction occurred in the case of *NOTCH3.*

### 2.5. DLK2 Modulates Phosphorylation of ERK1/2 MAPK, AKT and rpS6 Kinases in MDA-MB-231 Cells

**Figure 5:**
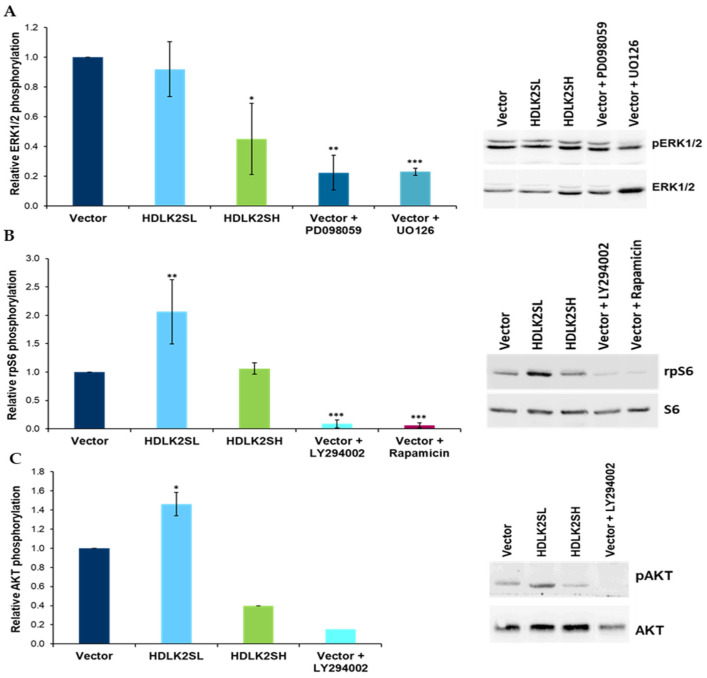
**Phosphorylation levels of ERK1/2 MAPK, rpS6 and AKT kinases in MDA-MB-231 cells overexpressing DLK2.** Representative Western blot analyses of phosphorylation levels of ERK1/2 MAPK (**A**), rpS6 (Ser235/236) (**B**), and AKT (Ser473) (**C**) in HDLK2SL and HDLK2SH cell pools. MDA-MB-231 cells stably transfected with the empty vector and treated with 10 μM of the MEK inhibitor U0126, or with 30 μM of the MAPKK inhibitor PD098059 for 24 h were used as controls for the analysis of the ERK1/2 MAPK phosphorylation. MDA-MB-231 cells stably transfected with the empty vector and treated with 10 μM of the PI3-K inhibitor LY294002 for 24 h was used as a control for the analysis of the rpS6 or AKT phosphorylation. MDA-MB-231 cells stably transfected with the empty vector and treated with 1 μg/mL of the mTOR inhibitor Rapamycin for 24 h were used as a control for the analysis of the rpS6 phosphorylation. * *p* ≤ 0.05, ** *p* ≤ 0.01, *** *p* ≤ 0.001 (Student’s *t*-test results relative to empty-vector cells).

The ERK/MAPK pathway appears to be constitutively activated in MDA-MB-231 cells, which likely is important in preventing anoikis and maintaining the anchorage-independent growth of these cells [29]. In a previous work, and in agreement with other reports [30,31,32], we showed that, in addition to NOTCH signaling, DLK1 inhibited the ERK1/2 MAPK signaling pathway in MDA-MB-231 cells [10]. Therefore, it was important to study whether DLK2 overexpression would also modify the steady state of ERK1/2 MAPK activation in these cells. To this end, we analyzed the phosphorylation status of ERK1/2 MAPK in our pools of DLK2-transfected cells by Western blot analysis. For HDLK2SL cells, no changes were observed relative to empty-vector cells, whereas HDLK2SH cells showed a significant decrease in ERK1/2 MAPK phosphorylation. As a control, we treated cells transfected with an empty vector with 10 μM of the MEK inhibitor U0126, or with 30 μM of the MAPKK inhibitor PD098059 for 24 h to inhibit ERK1/2 MAPK phosphorylation (Figure 5A).

Consistent with the previous results, we demonstrated that the phosphorylation levels of rpS6, a substrate for ERK1/2 MAPK, were significantly decreased when DLK1 was overexpressed [10]. Accordingly, we studied the phosphorylation levels of rpS6 in our DLK2-transfected cells. In this case, however, we observed different results. HDLK2SL cells showed a significant increase in rps6 phosphorylation levels, whereas no changes were observed in HDLK2SH cells with respect to empty-vector cells (Figure 5B). Here, as a control, we treated empty-vector-transfected cells with 10 μM of the PI-3 kinase inhibitor LY294002, or with 1 μg/mL of the mTOR kinase inhibitor, Rapamycin.

Finally, the PI3K/AKT pathway, an important signaling pathway related to cell quiescence and proliferation, which is activated in 30–40% of breast cancer cases [33], was not modified in MDA-MB-231 cells transfected with DLK1 [10]. However, HDLK2SL cells showed a significant increase in the activation of this pathway, whereas HDLK2SH cells showed a decrease (Figure 5C). As a control, we treated empty vector-transfected cells with 10 μM of the PI-3 kinase inhibitor LY294002 to inhibit PI3K/AKT activation.

### 2.6. DLK2 Overexpression Affects MDA-MB-231 Tumor Development In Vivo

**Figure 6:**
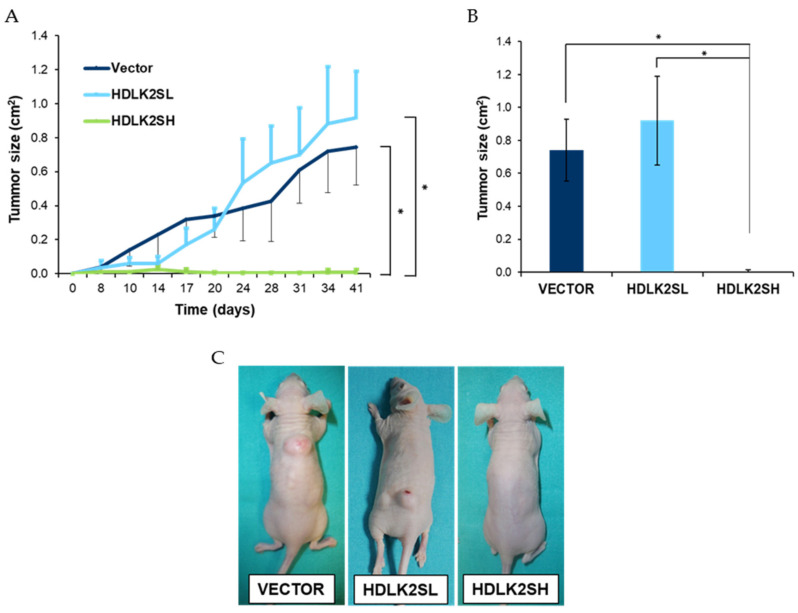
**High levels of DLK2 expression in MDA-MB-231 cells inhibit tumor growth in vivo.** MDA-MB-231 cells from HDLK2SL- and HDLK2SH-transfectant pools or MDA-MB-231 cells stably transfected with the empty vector were subcutaneously injected into nude mice. (**A**) Tumor size was measured as described in Material and Methods section at the indicated time points. Results are represented as means ± SD. (**B**) The graph shows the means ± SD of the tumor sizes at the end of the experiment. * *p* < 0.05 (Mann–Whitney U test results for mice injected with the empty-vector cells). (**C**) Mice were euthanized after 41 days and photographed. Representative photographs of subcutaneous tumors formed in mice from each group are shown.

In our previous work [10], we observed that the significant effects exerted by DLK1 on the cellular and molecular events of MDA-MB-231 cells, which impacted their growth properties in vitro, also impacted their ability to form tumors in vivo. This ability was dependent upon DLK1 expression levels. Low levels of DLK1 expression accelerated tumor growth in vivo, whereas high levels of DLK1 expression caused tumors that grew more slowly than normal. To study the effects of different DLK2 expression levels on tumor growth in vivo, we subcutaneously injected MDA-MB-231 cells transfected with empty-vector cells, or HDLK2SL or HDLK2SH cell pools into nude mice and measured their growth rate for six weeks. The results were striking. While HDLK2SL cells showed a non-statistically significant increase in tumor growth compared to empty-vector cells, HDLK2SH cells completely failed to establish tumors in vivo (Figure 6A,B). Furthermore, in mice injected with HDLK2SL cells, a second smaller tumor appeared near the injection site (Figure 6C). These results indicate that different levels of DLK2 expression could have different consequences on the establishment and growth of MDA-MB-231 tumors.

## 3. Discussion

In a previous work, we reported that different levels of DLK1 expression exerted a significant modulation on the growth and tumorigenic properties of MDA-MB-231 triple-negative breast cancer cell line [10]. The present work aimed to study the effects exerted by DLK2, a protein homologous to DLK1, in the tumorigenic properties of this cell line [14]. As with DLK1, DLK2 was shown to inhibit NOTCH receptor signaling [8,11,13] and affect the outcome of several differentiation processes, in particular adipogenesis [8,10,13,34] and chondrogenic differentiation [35]. Therefore, it was important to study the effects of different levels of DLK2 expression on the growth of MDA-MB-231 breast cancer cells and their tumorigenic properties, and then compare them with the effects exerted by DLK1 in these cells, especially since NOTCH signaling was found to be a hallmark of triple-negative breast cancer tumors [36].

The effects on the growth and invasion capabilities of MDA-MB-231 cells observed upon the reconstitution of DLK1 [10] or upon DLK2 overexpression, are consistent with the role of NOTCH signaling in the growth of triple-negative breast cancer cells. Our data indicate that, as was the case with DLK1, the in vitro and in vivo growth properties of MDA-MB-231 cells are negatively affected by elevated levels of DLK2 expression (Figure 2 and Figure 6). High levels of DLK2 expression affected cell distribution throughout the cell cycle phases (Figure 2B). These changes were related to a significant increase in the expression of *CDKN1A* (p21, Cip1), a cyclin-dependent kinase inhibitor capable of inhibiting all cyclin/CDK complexes, and a downregulation of *CCND1* (cyclin D1) expression, which could result in the decreased cell growth that we observed in vitro and in vivo. The effects we detected on proliferation could also be related to the fact that high levels of DLK2 expression also increased apoptosis, as determined by annexin V/ propidium iodide assays. On the other hand, low levels of DLK2 overexpression led to increased cell proliferation, and changes in the cellular distribution throughout the cell cycle phases and the expression levels of p21, Cip1 and cyclin D1. In addition, cells with low levels of DLK2 expression showed a decreased apoptosis.

One possibility that may help to explain the similar effects caused by the overexpression of DLK1 and DLK2, despite the observed differences, is that both proteins were shown to act as inhibitors of NOTCH signaling in several cellular systems [6,7,8,9,10,11,12,13]. The data presented here confirm that DLK2 is also an inhibitor of NOTCH activation in MDA-MB-231 cells. The behavior of DLK2 as an inhibitor of NOTCH signaling in MDA-MB-231 cells closely mimics the behavior of DLK1 [10]. Both proteins inhibit NOTCH1 activation in a dose-dependent manner, as elevated levels of DLK1 or DLK2 expression correlate with the high levels of inhibition of NOTCH1 activation. In both cases, the rate of inhibition of NOTCH1 activation obtained by a high expression of DLK1 or DLK2 is similar to that obtained by treatment with 10 µM DAPT, an inhibitor of the γ−secretase complex that processes NOTCH receptors (Figure 4B). The similar behavior of both DLK proteins as inhibitors of NOTCH activation is also revealed by analyzing their effects on the expression levels of known target genes used as markers of NOTCH signaling levels. These genes include *HEY1*, *HEY2* and *HES1*. The overexpression of DLK2 generally led to decreased expression levels of *HEY1* and *HEY2*, but significantly increased expression levels of HES1 (Figure 4C) [10]. While the decrease in *HEY1* and *HEY2* expression levels was expected, the increase in *HES1* expression level was contrary to expectations. We do not have an explanation for this unexpected result. Although it is known that *HES1* expression levels can fluctuate widely [28], it is unlikely that the consistent effect of DLK2 overexpression on *HES1* expression levels in MDA-MB-231 cells is due to these fluctuations. These and previous data reveal that, in some cells or some contexts, the inhibition of NOTCH signaling can lead to the increased expression of some target genes, suggesting the existence of feedback mechanisms that may be altered by abrupt changes in NOTCH activation levels [8,13].

Despite all of the similar effects of DLK1 and DLK2 overexpression on MDA-MB-231 cells, there are some important differences, especially in the effects caused by both proteins on *NOTCH* mRNAs expression levels. In this regard, low or high levels of DLK2 expression caused a decrease in the expression levels of all *NOTCH* genes, except *NOTCH2* (Figure 4E).

Despite being able to inhibit overall NOTCH signaling levels to similar degrees, it is likely that DLK1 and DLK2 do not inhibit this signaling pathway with the same specificity. Most likely one of the proteins inhibits one of the NOTCH receptors preferentially to the others. This different specificity is reflected in different NOTCH-dependent genes being transcribed or silenced or in the same genes being transcribed to different degrees. Thus, each DLK protein influences the differentiation outcome and growth properties of the cells in a particular way. In this regard, it is worth mentioning that *NOTCH4* appears to be the most important *NOTCH* gene for inhibiting breast cancer growth, since the overexpression of NOTCH4 increases, whereas the inhibition of NOTCH4 reduces, the proliferation and invasiveness of triple-negative breast cancer cells [37]. Our data indicate that *NOTCH4* is one of the genes whose expression is more strongly reduced by high levels of DLK2.

On the other hand, NOTCH3 increases the establishment of tumors and metastasis [38,39]. However, the opposite effects are observed in vivo, since HDLK2SH cells were totally unable to establish tumors in nude mice (Figure 6).

DLK2 also affects the growth of MDA-MB-231 cells. The effects depend on DLK2 expression levels and the degree of inhibition of NOTCH signaling, as elevated DLK2 expression levels, which resulted in high levels of inhibition of NOTCH activation, produced a decreased cell growth and increased apoptosis. Meanwhile, low DLK2 expression levels, which resulted in low levels of the inhibition of NOTCH signaling, produced increased cell growth and decreased apoptosis. This dose-dependent inhibition of NOTCH activation by DLK proteins was also described in SK-MEL-2 melanoma cells, where it is also associated with an opposite effect on cell proliferation [9]. In this regard, previously published data showed that different levels of NOTCH signaling led to different phenotypic responses, including stimulatory or suppressive growth effects in mammary epithelial cells [40]. The effect we observed in MDA-MB-231 cells expressing high DLK2 levels is similar to that reported in other studies performed in the same cells with monoclonal antibodies that inhibit NOTCH1 activation. The treatment of cells with these antibodies resulted in the decreased cell proliferation and increased induction of apoptosis [41]. Nevertheless, it was recently reported that the overexpression of DLK2 (EGFL9) protein does not affect the proliferation of mammary epithelial cell, and that the downregulation of its expression does not affect the proliferation of mouse 4T1 and human SUM159 metastatic breast cancer cell lines [16].

We also observed that the overexpression of DLK2 in MDA-MB-231 cells resulted in gene expression changes related to invasiveness. Thus, the expression levels of the *SNAI2* gene were affected by DLK2 overexpression. The Slug (*SNAI2)* factor enhances the epithelial-to-mesenchymal transition by modulating E-cadherin and N-cadherin expression and shows antiapoptotic activity [42]. However, our results (Figure 3) show a somewhat contradictory situation. Low levels of DLK2 expression (HDLK2SL) produced increased levels of *SNAI2* expression, which is associated with increased levels of E- and N-cadherin expression. However, high levels of DLK2 expression (HDLK2SH) resulted in decreased levels of *SNAI2* expression, which is associated with even higher levels of E-cadherin expression and similar levels of N-cadherin expression than HDLK2SL cells (Figure 3B,C). Therefore, regardless of the changes in *SNAI2* expression caused by the different levels of DLK2 expression, E-cadherin and N-cadherin expression is increased. The increase in the level of E-cadherin expression is, in fact, correlated with the DLK2 expression levels of the transfected MDA-MB-231 cells. However, changes in the invasiveness properties of the cells are not consistent with this, since despite the increased levels of E-cadherin and N-cadherin expression in HDLK2SL cells relative to empty-vector cells, an increased invasiveness is observed. This effect is different from what occurs in HDLK2SH cells that do not show significant changes in invasiveness (Figure 3A,C) compared to empty-vector cells. In this regard, previously published data show that the inhibition of NOTCH signaling led to an increase in E-cadherin expression and a decrease in the invasive capacity of MDA-MB-231 cells [43], which agrees with our findings showing that HDLK2SH cells exhibit a high rate of inhibition of NOTCH1 activation and prevent tumor development in nude mice.

Strikingly, despite the observed differences in the expression levels of slug and N-cadherin and E-cadherin caused by DLK2 overexpression in MDA-MB-231 cells, similar effects on invasiveness features were observed for MDA-MB-231 cells overexpressing different levels of DLK1 [10]. This suggests that, in addition to their effect on *SNAI2* and *CDH* gene expression, both DLK1 and DLK2 may affect other shared mechanisms that modulate the invasive properties of MDA-MB-231 and other cancer cells. DLK1 is known to affect MMP9 expression levels through NOTCH signaling to promote lung cancer cell invasion [44]. Recently, it was also described that the ectopic expression of (DLK2) EGFL9 significantly promotes cell migration and invasion in mammary epithelial cell lines and cancer metastasis in vivo, while a knockdown of DLK2 in highly metastatic breast cancer cell lines decreased cell migration and invasion and inhibited distant tumor metastasis in vivo [16]. These effects appear to be the result of the physical interaction between DLK2 and cMET receptor. This interaction activates the cMET receptor and downstream signaling pathways that are involved in cell migration and invasion. It is possible that different levels of DLK2 expression may led to different effects on MDA-MB-231 cells depending on the degree of DLK2 interaction with cMET or NOTCH, as it was reported that NOTCH1 and cMET may crosstalk with each other and are involved in a complex feedback loop [45,46].

Moreover, Jagged1, a canonical ligand of NOTCH receptors, was reported to modulate epithelial-to-mesenchymal transition in breast cancer cells through NOTCH signaling, increase the expression levels of *SNAI2* and decrease those of *CDH1.* Jagged1 also modulates cell proliferation, as the inhibition of Jagged1 expression in MDA-MB-231 cells is sufficient to reduce cell cycle progression [5,47]. We observed that increasing DLK2 expression levels in MDA-MB-231 cells led to a proportional and significant decrease in *JAG1* expression (Figure 4D). These and previous data argue strongly in favor of DLK1 and DLK2 acting upon common molecular mechanisms to modulate NOTCH activation, which in turn may be affected by the decreased expression of at least one of the most important NOTCH canonical ligand gene, such as *JAG1*. However, our data indicate that *JAG1* expression levels do not directly correlate with the invasiveness of DLK2-overexpressing cells. Only MDA-MB-231 cells, with high levels of DLK2 expression that have decreased levels of *JAG1* expression, correlate with reduced cell cycle progression and invasiveness in vivo.

We also analyzed the effects of different levels of DLK2 expression on other signaling pathways already known to be affected by DLK1 expression [10] and NOTCH signaling [5], which ultimately affect gene expression and cell behavior in breast cancer cells [48]. The ERK1/2 MAPK pathway, involved in cell proliferation and migration, as well as epithelial-to-mesenchymal transition, is reported to crosstalk with the NOTCH1 signaling pathway in MDA-MB-231 cells [49]. DLK2 behaved similarly to DLK1 in the regulation of ERK1/2 MAPK phosphorylation. The overexpression of DLK2 led to the decreased phosphorylation of ERK1/2 MAPK, although only high DLK2 expression led to a significant inhibition of ERK1/2 MAPK phosphorylation (Figure 5A) [10]. The activation of NOTCH1 signaling was described as leading to increased ERK1/2 MAPK activity, which in turn led to increased expression levels of *JAG1* in MDA-MB-231 cells [50]. The inhibition of ERK1/2 MAPK caused by the increased expression levels of DLK2 may help explain the inhibition of *JAG1* in our transfectant cells.

The mTOR pathway plays a role in breast cancer cell proliferation and anti-cancer drug resistance and was also reported to crosstalk with the NOTCH signaling pathway in several malignant cells, including breast cancer cells [51]. The phosphorylation status of rpS6 kinase differed between MDA-MB-231 cells transfected with DLK1 or DLK2. In this case, low levels of DLK2 expression caused an increase in rpS6 phosphorylation levels, and high levels of DLK2 expression did not significantly affect rpS6 phosphorylation (Figure 5B) [10]. Additionally, NOTCH can activate AKT kinase signaling, which is involved in the growth, proliferation, motility, and survival of breast and other cancer cells [5,51]. The overexpression of DLK2 in MDA-MB-231 cells also results in changes in the level of AKT phosphorylation, which again appears to be dependent on DLK2 expression levels. In this case, low DLK2 expression levels cause an increase in AKT phosphorylation levels, whereas high DLK2 expression levels cause a decreased in AKT phosphorylation levels. These data suggest that the growth and invasion capabilities of MDA-MB-231 cells may depend on the level of activation of NOTCH signaling and/or the level of phosphorylation of ERK1/2 MAPK, rpS6 and PI3K/AKT kinases.

The data presented here, and those already published on the effect of DLK1 in MDA-MB-231 cells [10], reveal that both proteins are able to inhibit NOTCH signaling and modulate other signaling pathways affecting the growth and invasive properties of MDA-MB-231 cells in vitro and in vivo. Furthermore, our data show that the level of activation of NOTCH and the degree of phosphorylation of the kinases studied in this work, which appear to be modulated by the expression levels of DLK2, may lead to opposite effects on the features of MDA-MB-231 breast cancer cells. In addition, we revealed that the insufficient inhibition of NOTCH signaling may enhance tumor cell growth, which should be considered for future treatment strategies. To our knowledge, studies on the possible stronger effects of the combined activity of DLK1 and DLK2 on breast cancer cell growth and/or their invasive properties have not yet been performed. Thus, further studies are warranted to explore this possibility and could open a new potential therapeutic window using both proteins as therapeutic tools against this aggressive triple-negative form of breast cancer.

## 4. Materials and Methods

### 4.1. Plasmids

The HDLK2S plasmid contains the full-length human *DLK2* cDNA (MGC Full-Length clone IMAGE ID 54954558) cloned into the *Hin*dIII-*Not*I restriction sites of the pLNCX2 expression vector [9].

### 4.2. Cell Culture and Transfection Conditions

The human breast cancer cell line MDA-MB-231 was purchased from the American Type Culture Collection (HTB-26). Cells were cultured at 37 °C in a humidified atmosphere at 5% CO_2_, with a 1:1 mixture of DMEM (Dulbecco’s modified Eagle’s medium, Lonza, Pontevedra, Spain) and F12 (Lonza) cell culture media, containing 10% FBS (Fetal bovine serum) (Lonza). Transfections were performed in 80% confluent cells, using FUGENE HD Transfection Reagent and plasmid DNA in a 3:1 ratio, following the manufacturer’s recommendations (Roche Diagnostics GmbH, Mannheim, Germany). Stable transfectants were selected under standard culture conditions in a selective medium containing G418 (Sigma, Madrid, Spain) at 500 μg/mL. In some experiments, cells were treated for 24 h with 10 μM γ−secretase IX inhibitor (DAPT: N-[N-(3,5-difluorophenacetyl)-L-alanyl]-S-phenylglycine t-butyl ester) (Calbiochem, Madrid, Spain), 30 μM PD098059 (MEK inhibitor, New England Biolabs, Inc., Barcelona, Spain), 10 μM UO126 (MEK inhibitor, Sigma), 10 μM LY294002 (PI-3 kinase inhibitor, Sigma), 1 μg/mL Rapamycin (mTOR kinase inhibitor, Sigma), or solvent DMSO as control.

### 4.3. Cell Growth Assays

Cell proliferation was measured by MTT (3-(4,5-dimethylthiazol-2-yl)-2,5-diphenyltetrazolium bromide) assays for 8 days, following the protocol provided by the manufacturer (Sigma). A total of 1500 cells per well were seeded in a 96-well plate, and the culture medium was replaced every two days. Proliferation assays were carried out for eight days. These assays were repeated at least three times.

### 4.4. Cell Cycle Assays

Stably transfected MDA-MB-231 cells (1 × 10^5^ cells/mL) were seeded into a 10 cm dish. After 24 h, cells were incubated with 10 μM bromodeoxyuridine (BrdU) (APC BrdU Flow Kit, BD Pharmingen, Madrid, Spain) for one hour. Cells were harvested, fixed and permeabilized in BD Cytofix/Cytoperm Buffer (APC BrdU Flow Kit, BD Pharmingen) for 30 min. The cells were then washed with BD Perm/Wash Buffer (APC BrdU Flow Kit, BD Pharmingen) and centrifuged (180× *g*, 5 min). Then, cells were resuspended with BD Cytoperm Plus Buffer, washed in BD Perm/Wash Buffer, incubated with BD Cytofix/Cytoperm Buffer, washed again in BD Perm/Wash Buffer, and incubated in DNase solution (300 μg/mL) (APC BrdU Flow Kit, BD Pharmingen) for 1 h at 37 °C.

DNase-treated cells were washed by adding BD Perm/Wash Buffer, resuspended in BD Perm/Wash Buffer containing diluted (1:50) fluorescent anti-BrdU antibodies (APC BrdU Flow Kit, BD Pharmingen), and incubated for 20 min at room temperature. Finally, cells were washed with BD Perm/Wash Buffer and resuspended in 20 μL of 7-amino-actinomycin D solution (APC BrdU Flow Kit, BD Pharmingen) in the dark. Stained cells were resuspended in staining buffer (1xDPBS + 0.03% FBS + 0.09% sodium azide) and analyzed on a FACSCanto II cell analyzer (BD Biosciences, Madrid, Spain). The percentage of cells in each cell cycle phase (G0/G1, S or G2/M) was calculated by using FACSDiva v 6.1.3 software (BD Biosciences). These assays were repeated at least three times.

### 4.5. Annexin V Assay

Stably transfected MDA-MB-231 cells were incubated in the absence of serum for 24 h. Cells were then washed twice with cold PBS (Phosphate Buffered Saline, Lonza) and resuspended in 1X Annexin V Binding Buffer (FITC (fluorescein isothiocyanate) Annexin V Apoptosis Detection Kit I, BD Pharmigen) at a concentration of 1 × 10^6^ cells/mL. One hundred microliters (1 × 10^5^ cells) were transferred to a 5 mL culture tube and incubated in the dark with 5 μL of FITC-annexin V and 5 μL propidium iodide for 15 min, following the manufacturer´s protocol (FITC Annexin V Apoptosis Detection Kit I, BD Pharmigen). Finally, 1X Annexin V Binding Buffer (400 μL) was added and the cells were analyzed by flow cytometry (FACSCanto II, BD Biosciences).

### 4.6. Invasion Assays

In vitro cell invasion assays were performed in Matrigel BD BioCoat Invasion Chambers (BD Biosciences), following the manufacturer´s recommendations. Briefly, 1 × 10^5^ transfected cells, resuspended in 500 μL of serum-free medium, were plated in the upper chamber, which was introduced into the lower chamber containing the complete medium. Chambers were incubated for 24 h at 37 °C, in a humidified atmosphere at 5% CO_2_. Non-invasive cells were removed by wiping the top of the membrane, and invasive cells were fixed and stained. Cell invasion through the membranes was determined by microscopically counting cells present in six random fields at 100× magnification. These assays were repeated at least three times.

### 4.7. RNA Extraction and RT-qPCR

For gene expression analysis, cell monolayers were washed twice with PBS and detached with Trypsin/Versene (Lonza). Cells were then collected by centrifugation (180× *g*, 5 min, at 4 °C) and washed twice with PBS. Total RNA was isolated by using the RNeasy Kit (Qiagen, Valencia, Spain). After this, DNase treatment (Qiagen), RNA (1 μg) was reverse transcribed by using the cDNA RevertAidH Minus First Strand kit (Fermentas, Madrid, Spain), according to the manufacturer’s recommendations. To perform RT-qPCR, cDNA was amplified by PCR using the SYBR-GREEN Master Mix and StepOnePlus Real-Time PCR System (Applied Biosystems, Madrid, Spain). The PCR conditions used were an initial denaturation step at 95 °C, followed by 30 s at 60 °C. *GAPDH* expression was used as a control to compare the CT of the different samples. The primers used to determine the expression level of *GAPDH*, *DLK2*, *HEY1*, *HEY2*, *HES1*, *NOTCH1*, *NOTCH2*, *NOTCH3*, *NOTCH4*, *JAG1*, *CCND1*, *CDKN1A*, *SNAI2*, *CDH2* and *CDH1* were previously described [10].

### 4.8. Western Blot

For protein expression studies, cell pellets were lysed in RIPA buffer containing a 100-fold dilution of Phosphatase Inhibitor Cocktails 1 and 2 (Sigma), incubated on ice for 30 min and centrifuged at 10,000× *g* for 10 min at 4 °C. The protein content of cleared lysates was quantified, after which 100 μg of total protein extract was loaded on 12% (*w*/*v*) SDS-PAGE (sodium dodecyl sulfate-polyacrylamide gel electrophoresis) gels. Western blotting was performed as previously described [6] by using the following antibodies: anti-human DLK2 (Proteintech, Manchester, United Kingdom), diluted 1:500 in 5% *w*/*v* Bovine Serum Albumin (BSA), 1X TBS, 0.1% Tween^®^ 20 (TBS-T); anti-cleaved NOTCH1 (Val 1744, Cell Signaling, Barcelona, Spain), diluted 1:500 in 5% *w*/*v* BSA, TBS-T; anti-pERK1/2 (E-4, Santa Cruz Biotechnology, Inc., Heidelberg, Germany) diluted 1:500 in 1% *w*/*v* nonfat dry milk, TBS-T, and anti-ERK2 (C-14, Santa Cruz Biotechnology, Inc.), diluted 1:1000 in 1% *w*/*v* nonfat dry milk, TBS-T; anti-phospho AKT (Ser473, Cell Signaling), diluted 1:500 in 5% *w*/*v* BSA, TBS-T and anti-AKT, diluted 1:1000 in 5% *w*/*v* BSA, TBS-T (Cell Signaling); anti-phospho rpS6 (Ser235/236, Cell Signaling), diluted 1:1000 in 5% *w*/*v* BSA, TBS-T; anti-rpS6, diluted 1:1000 in 5% *w*/*v* BSA, TBS-T (Cell Signaling); and anti-alpha tubulin (Sigma), diluted 1:5000 in 5% *w*/*v* nonfat dry milk, TBS-T. Western blot images were obtained by using a Fujifilm LAS-3000 Imager analyzer or by developing exposed films (CP-BU New) in a Curix 60 developing apparatus (AGFA, Barcelona, Spain). Densitometric analyses of Western blot signals were made by using Quantity One 1D analysis software (Bio-Rad, Madrid, Spain).

### 4.9. Luciferase Assays

To estimate NOTCH-dependent transcriptional activity, MDA-MB-231 cells were co-transfected with the pGLucWT (CSL/RBP-Jk-LUC) plasmid and the pLNCX2 (empty vector) or DLK2 expression (HDLK2S) plasmids. The pGLucWT plasmid contains the luciferase reporter gene under the control of a promoter with four copies of a CSL/RBP-Jk (recombining binding protein suppressor of hairless) binding site [6]. To measure the inhibition of NOTCH-dependent transcriptional activity caused by DAPT, cells were co-transfected with pLNCX2 and pGLucWT plasmids and incubated in the presence of 10 μM DAPT for 24 h. To measure luciferase activities, cells were lysed and processed by using the dual luciferase kit (Dual-Luciferase Reporter Assay System, Promega, Madrid, Spain) 24–48 h after transfection, following the manufacturer’s recommendations. To normalize the data obtained, cells were also transfected with pRLTK (renilla expression plasmid). Luciferase and renilla activities were measured on a Monolight 3096 Microplate Luminometer (Becton Dickinson). These assays were repeated at least three times.

### 4.10. Animal Studies

Female nude athymic-Foxn1nu mice (5-week-old) were supplied by Harlan Laboratories (Barcelona, Spain). Stably transfected MDA-MB-231 cells (2.5 × 10^6^ cells resuspended in 200 μL of PBS) were injected subcutaneously into the dorsal flanks of the mice for a total of 5 mice per group. Mice were sacrificed 41 days after injection. Tumor size was calculated by the following formula: length (mm) × width (mm). In vivo experiments were performed in accordance with Spanish and European regulations and were approved by the Animal Care and Use Committee of the University of Castilla-La Mancha.

### 4.11. Statistical Analysis

Data are presented as the mean ± S.D of at least three independent assays. Data were also analyzed with the GraphPad and/or SPSS software packages for two-tailed Student’s *t*-test or Mann–Whitney U test to determine statistical significance in relation to cells transfected with pLNCX2 (empty vector). A *p* value ≤ 0.05 was considered statistically significant (*); a *p* value ≤ 0.01 was considered highly statistically significant (**); and a *p* value of ≤0.001 was considered extremely statistically significant (***).

## 5. Conclusions

Our data revealed for the first time that different levels of DLK2 expression in MDA-MB-231 breast cancer cells can produce opposite effects on tumor invasiveness and growth rate both in vitro and in vivo. These opposite effects are most likely due to the induction of different levels of NOTCH signaling and ERK1/2, AKT and mTOR kinases phosphorylation. Low levels of DLK2 expression resulted in a slight inhibition of NOTCH signaling activation, the increased phosphorylation of rpS6 and AKT kinases, and increased invasiveness and cell growth in vivo and in vitro. Conversely, high levels of DLK2 expression produced a strong inhibition of NOTCH signaling, decreased the phosphorylation of ERK1/2 MAPK and AKT kinases, decreased cell growth rate in vitro and failed to establish tumors in vivo.

**Figure 7:**
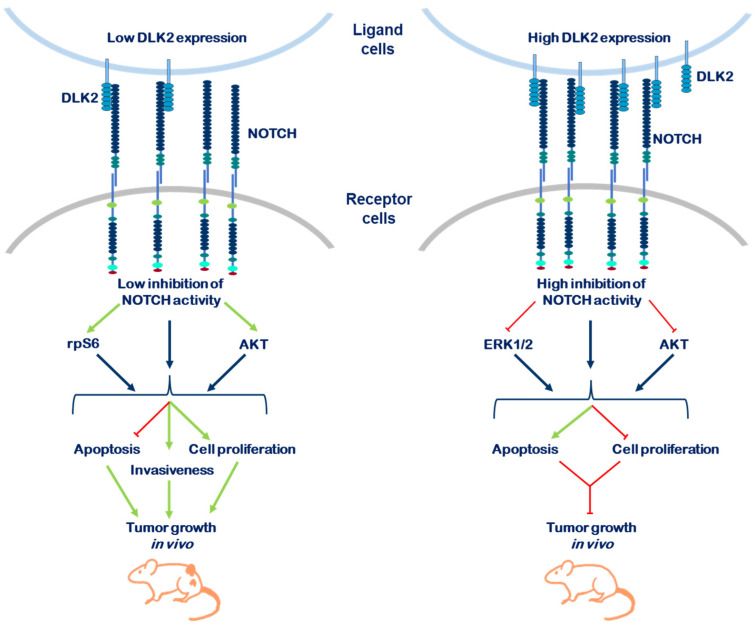
**DLK2 inhibits NOTCH activation in a dose-dependent manner and affects the features of MDA-MB-231 breast cancer cells.** Different levels of DLK2 expression in MDA-MB-231 cells can produce opposite effects on cell proliferation and apoptosis, invasion properties and tumor growth in nude mice, most likely by inducing different levels of NOTCH activation and the phosphorylation of ERK1/2, AKT and mTOR kinases.

The data presented in this work and in our previously published work reveal that both DLK proteins are capable of inhibiting NOTCH signaling and modulating kinase signaling pathways involved in cancer. Most likely, the level of NOTCH activation, modulated by DLK1 and DLK2, may led to opposite effects on the invasive properties of MDA-MB-231 breast cancer cells and tumor cell growth in vitro and in vivo. Furthermore, we revealed that the insufficient inhibition of NOTCH signaling may enhance tumor cell growth. This aspect should be considered for patient treatment strategies against this aggressive form of breast cancer. Figure 7 summarizes the effects of the levels of DLK2 expression on MDA-MB-231 cell growth and invasion properties revealed in this work.

## Data Availability

All data are available within the main body of the manuscript.
